# P-26. Increasing HPV Vaccination Rates in an Underserved Urban Population

**DOI:** 10.1093/ofid/ofae631.233

**Published:** 2025-01-29

**Authors:** Heather M Wang, Shirin Voss, Divya Viswanathan, Nikita Tangella, Piyumika de Silva, Jared Walsh

**Affiliations:** Rutgers NJMS, Newark, New Jersey; Rutgers NJMS, Newark, New Jersey; Rutgers NJMS, Newark, New Jersey; Rutgers NJMS, Newark, New Jersey; Rutgers NJMS, Newark, New Jersey; Rutgers NJMS, Newark, New Jersey

## Abstract

**Background:**

Uninsured individuals are found to have lower rates of human papillomavirus (HPV) vaccination initiation compared to insured patients. Our primary care clinic is based in an urban setting, and is primarily comprised of non-English-speaking and uninsured individuals. Previous review of the clinic’s HPV vaccination rate showed 13% of patients were fully vaccinated against HPV and 24% were partially vaccinated, lower than the national rates of 21.5% and 39.5%, respectively. This study aimed to improve the rate of HPV vaccination to meet the national average.

**Figure:**
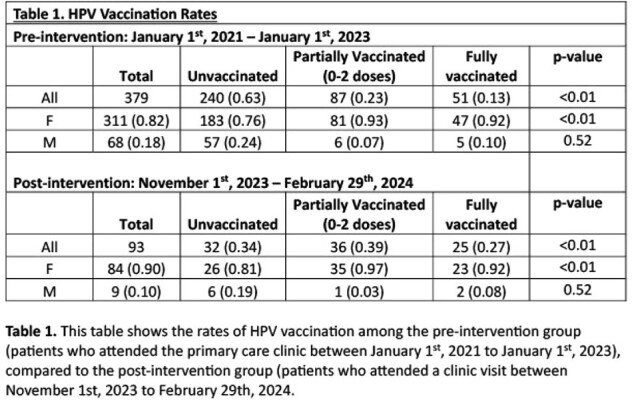

**Methods:**

This retrospective chart review evaluated all males age 18-21 and females age 18-26 who attended a primary care visit at our clinic between January 1^st^ 2021 to February 29^th^ 2024. A quality improvement initiative was implemented from January 1^st^, 2023 to November 1^st^, 2023, and included educating the medical team, crafting informational pamphlets, and fostering discussions with patients. HPV vaccination rates were examined pre-intervention (January 1^st^, 2023-January 1^st^, 2023) and post-intervention (November 1^st^, 2023 to February 29^th^, 2024.

**Results:**

379 patients were included in the pre-intervention group: 311 (82%) female, 68 (18%) male, and 93 patients were included post-intervention: 84 (90%) female and 9 (10%) male. The rate of patients fully vaccinated against HPV increased from 13% (n=51/379) from the pre-intervention group compared to 27% (n=27/93) in the post-intervention group. The rate of unvaccinated patients decreased from 63% (n=240/379) to 34% (n=32/93). This change in vaccination rates was statistically significant (p< 0.01).

**Conclusion:**

Implementation of a quality improvement initiative to educate the medical staff and patients allowed for a statistically significant increase in the rate of HPV vaccination in an urban primary care practice. Continued collection of vaccination data from our clinic will allow for further guidance on how to target vaccine uptake in this population.

**Disclosures:**

**All Authors**: No reported disclosures

